# Kidney Shear Wave Speed Values in Subjects with and without Renal Pathology and Inter-Operator Reproducibility of Acoustic Radiation Force Impulse Elastography (ARFI) - Preliminary Results

**DOI:** 10.1371/journal.pone.0113761

**Published:** 2014-11-26

**Authors:** Flaviu Bob, Simona Bota, Ioan Sporea, Roxana Sirli, Ligia Petrica, Adalbert Schiller

**Affiliations:** 1 Department of Nephrology, “Victor Babes” University of Medicine and Pharmacy, Timişoara, Romania; 2 Department of Gastroenterology and Hepatology, “Victor Babes” University of Medicine and Pharmacy, Timişoara, Romania; Rensselaer Polytechnic Institute, United States of America

## Abstract

**Aim:**

to assess the inter-operator reproducibility of kidney shear wave speed, evaluated by means of Acoustic Radiation Force Impulse (ARFI) elastography, and the factors which influence it.

**Methods:**

Our prospective pilot study included 107 subjects with or without kidney pathology in which kidney shear wave speed was evaluated by means of ARFI elastography. Intraclass correlation coefficient (ICC) was used to assess ARFI elastography reproducibility.

**Results:**

A strong agreement was obtained between kidney shear wave speed measurements obtained by the two operators: ICC = 0.71 (right kidney) and 0.69 (left kidney). Smaller ICCs were obtained in “healthy subjects”, as compared to patients with kidney diseases (0.68 vs. 0.75), in women as compared with men (0.59 vs. 0.78), in subjects younger than 50 years as compared with those aged at least 50 years (0.63 vs. 0.71), in obese as compared with normal weight and overweight subjects (0.36 vs. 0.66 and 0.78) and in case of measurements depth <4 cm or >6 cm as compared with those performed at a depth of 4–6 cm from the skin (0.32 and 0.60 vs. 0.81).

**Conclusion:**

ARFI elastography is a reproducible method for kidney shear wave speed assessment.

## Introduction

Fibrosis is the final common pathway that leads to advanced stages of chronic kidney disease, irrespective of the underlying cause of the disease: glomerular disease, tubulo-interstitial disease or vascular disease. Renal biopsy, with all its potential complications, is the currently accepted method to assess renal fibrosis [1].

Therefore, it is important to search for new non-invasive methods to assess renal fibrosis. Today, elastographic techniques are used for the non-invasive staging of liver fibrosis. These methods can be classified as: qualitative (strain elastography) – Real Time Tissue-Elastography (Hi-RTE) (Hitachi, Japan) [2], [3] and quantitative (shear wave elastography), such as Transient Elastography (TE) by using a FibroScan device (Echosens, Paris, France) [4], [5], Acoustic Radiation Force Impulse (ARFI) elastography by using Virtual Touch Tissue Quantification application (Siemens AG, Erlangen, Germany) [6], [7] and 2D- shear wave elastography by using the Aixplorer device (SuperSonic Imagine S.A., Aix-en-Provence, France) [8], [9]. Because these elastographic methods are useful for a non-invasive assessment of fibrosis in the liver, they may also be used for the evaluation of kidney diseases.

ARFI elastography is based on the propagation of shear waves, which are progressively attenuated due to their absorption in the soft tissue, and the speed of propagation of the shear wave that is measured [6].

Unlike TE, ARFI elastography has the advantage of being integrated into an standard ultrasound system, and is used extensively in other tissues (breast, thyroid, gastrointestinal tract, prostate, muscles, etc) [10]–[14].

A pilot study performed by Arndt et al. [15] in kidney allografts, showed that renal parenchymal stiffness measured by TE reflects interstitial fibrosis. But considering the fact that the heterogeneous kidney morphology can affect measurements performed by TE [16], other methods were proposed to assess kidney shear wave speed in renal transplant recipients: ARFI elastography [17]–[20] and 2D- shear wave elastography (Aixplorer, SuperSonic Shear Imaging) [20], [21]. However, recent data show that it is possible that renal fibrosis is not the only factor influencing tissue stiffness at the level of the kidney. [22]–[24] It is possible that kidney shear wave speed measured using ARFI elastography is influenced by renal blood flow as well. [25], [26].

In order to validate these methods in renal diseases it is important to know if they are reproducible for different users. Thus, we aimed to assess in non-transplanted subjects the inter-operator reproducibility of kidney shear wave speed assessment by ARFI elastography, and the factors which influence it. Also, we studied the values of kidney shear wave speed in subjects with and without kidney diseases.

## Subjects and Methods

### Subjects

Our prospective study included subjects in which kidney shear wave speed was evaluated by means of ARFI elastography between November 2012–March 2013. Subjects with or without kidney pathology were included in our study. The subjects without kidney pathology (“healthy subjects”) were: healthy volunteers (medical students, nurses and medical doctors from our hospital) or patients hospitalized in various Departments of our Hospital. Healthy volunteers were subjects without a history of kidney diseases, without arterial hypertension, diabetes mellitus, but we did not perform additional tests, such as biological or urinary tests. All of them had a normal renal ultrasonography and the difference in length between the right and left kidney was less than 15 mm. The patients hospitalized in various Departments of our Hospital were defined as subjects without kidney pathology if they did not have a history of kidney diseases and the biological tests (serum creatinine and blood urea nitrogen) were normal and proteinuria and hematuria were absent. Also, these subjects had a normal renal ultrasonography and the difference in length between the two kidneys was less than 15 mm.

The patients with various acute or chronic kidney diseases were patients diagnosed and hospitalized in the Nephrology Department. Only patients with both kidneys present were included in our study.

All subjects included in our study signed informed consent; the study was approved by the local Ethics Committee and was in accordance with the Helsinki Declaration of 1975. The authors were the subjects shown in the images in this manuscript. All of authors and the local Ethics Committee approved the use of these images.

### Ultrasound examination

The ultrasound examination was performed in each subject in the same session with ARFI elastography measurements, using a Siemens Acuson S2000 ultrasound system (Siemens AG, Erlangen, Germany), with a convex array probe of 4–9 MHz (Siemens AG, Erlangen, Germany). The kidney length, the renal cortex thickness and kidney structure (echogenicity, presence of renal lithiasis, tumours or hydronephrosis) were reported.

### ARFI elastography

ARFI elastography was performed in all subjects with a Siemens Acuson S2000 ultrasound system by using Virtual Touch Tissue Quantification application, software version 2.0, by 2 operators: a gastroenterologist with 5 years experience in conventional ultrasound examination, more than 600 ARFI elastography measurements performed in the liver, spleen, thyroid and approximately 40 ARFI elastography measurements performed in the kidney before the start of this study; and a nephrologist with 10 years experience in conventional ultrasound examination and approximately 50 kidney shear wave speed measurements performed using ARFI elastography before this study.

**Figure 1:**
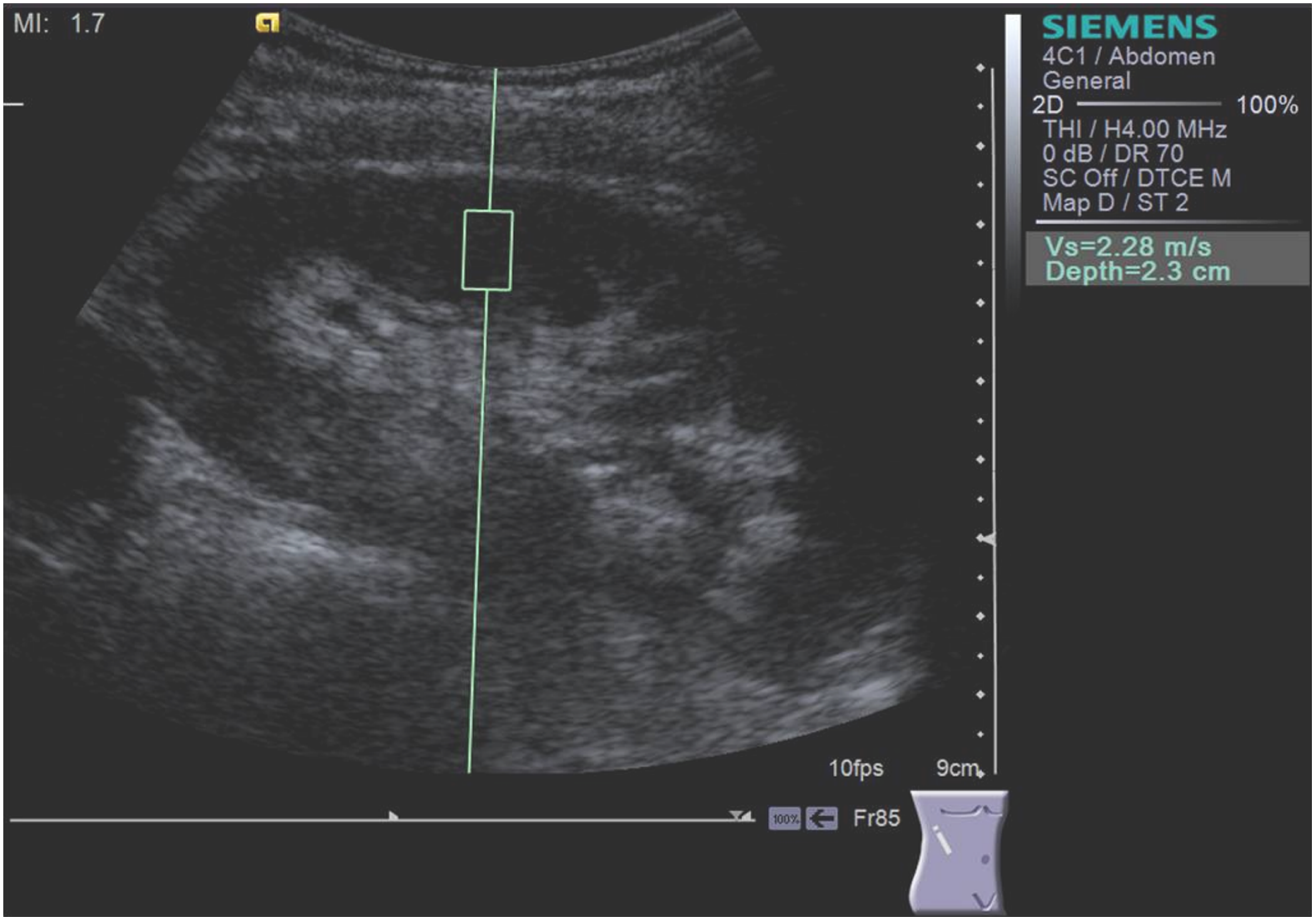
Kidney shear wave speed measured by ARFI elastography in the right kidney by the operator 1 (S.B.).

**Figure 2:**
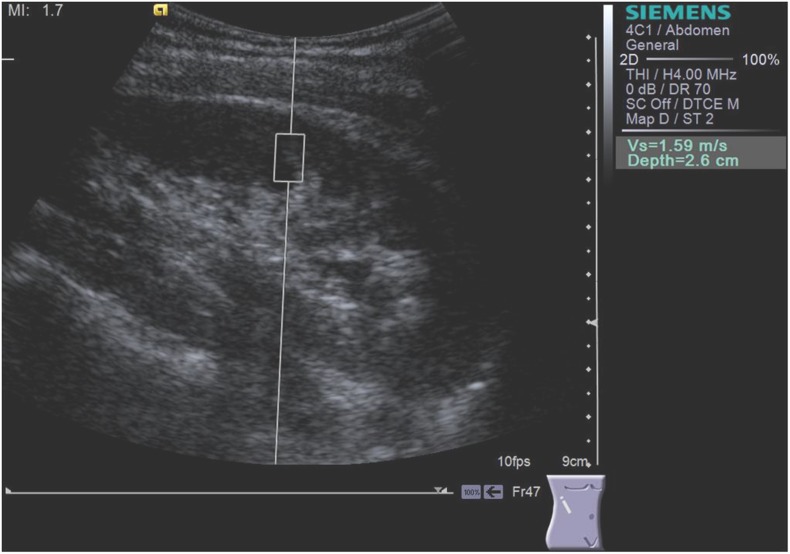
Kidney shear wave speed by ARFI elastography in the right kidney by the operator 2 (F.B.).

Scanning was performed with the patient in lateral decubitus. The “box” with a predefined size of 5 mm in width and 10 mm length was positioned by the operator in the mid-portion of the kidney, in the renal cortex, perpendicular to the kidney, while the patients were asked to stop breathing for a moment, in order to minimize breathing motion (Fig. 1, 2). Because it is known that tissue architecture, such as the high degree of anisotropy, has an impact on kidney shear wave speed values [25], we standardized the position of the patient during ARFI elastography. According to a recent study performed by our group the measurements should be performed in the mid portion of the renal parenchyma in lateral decubitus or prone position, according to the patient acoustic window [27]. Both operators performed the ARFI measurement by applying a minimal scanning pressure in order to reduce the variations due to pressure changes [28]. In each subject 5 valid measurements (meaning the measurements for which a numerical value of kidney shear wave speed was obtained) were performed for each operator and a median value was calculated, the result being expressed in meters/second (m/s). If the measurement was not valid, “X.XX” was displayed on the screen. The maximum depth at which ARFI elastography measurements can be performed is 8 cm [29], [30].

The kidney shear wave speed measurements were performed by the two operators in the same day (1–3 hours between measurements), the operators being blinded to the results of previous measurements and to the kidney pathology. Because, as already mentioned, renal blood flow has an influence on kidney shear wave speed, in all patients blood pressure has been measured before every assessment, and no difference greater then 10 mmHg has been found between the two measurements of every patient.

### Statistical analysis

The statistical analysis was performed using the MedCalc Software, version 12.4.0. (MedCalc program, Belgium). The distribution of the numerical variables was first tested by the Kolmogrov-Smirnov test. [31] In case of numerical variables with normal distribution (body mass index – BMI, kidney shear wave speed values) mean value and standard deviation (SD) were calculated, while in case of non-normal distribution (age) median values and range intervals were utilized. Qualitative variables were presented as numbers and percentages. To compare the kidney shear wave speed values obtained by the two operators parametric (paired t-test) was used, because the kidney shear wave speed values had normal distribution. The intraclass correlation coefficient (ICC) was used to assess the inter-operator reproducibility of kidney shear wave speed assessed by ARFI elastography. The ICC values were interpreted as: poor agreement (ICC = 0–0.20), fair agreement (ICC = 0.30–0.40), moderate agreement (ICC = 0.50–0.60), strong agreement (ICC = 0.70–0.80) and excellent agreement (ICC>0.80) [32], [33]. An ICC of 1 means that all variability relates to patient variability (patient effect) and that there is no variability related to the operator (operator effect) [32], [33]. As ICC decreases, the operator effect begins to predominate over patient effect. Inter-operator agreement was calculated as the agreement between the ARFI elastography kidney shear wave speed measurements of the two observers [32], [33]. In the statistical analysis the rater was considered as a random effect for the inter-operator agreement. 95% confidence intervals were calculated for all statistic tests. A p-value less than 0.05 was considered as statistically significant.

## Results

The kidney shear wave speed was assessed by means of ARFI elastography in 107 subjects. From these, 5 were excluded because they had only one kidney and 14 “healthy subjects” were excluded because the difference between the sizes of the two kidneys was over 15 mm. So, 88 subjects were included and evaluated in the final analysis. None of the subjects had renal lithiasis, tumours or hydronephrosis. The main characteristics of the subjects included in the study are presented in Table 1.

**Table 1:** The main characteristics of the subjects included in the study.

Parameter		
Age (years)		47 (19–83)
Gender:	-male	n = 40 (45.5%)
	-female	n = 48 (54.5%)
Body mass index (BMI) (kg/m^2^)		25.6±5.8
Kidneypathology:	- without kidney pathology	n = 68 (77.3%) (33 healthy volunteers and 35 hospitalized subjects)
	- acute pyelonephritis	n = 5 (5.7%)
	- acute kidney injury	n = 3 (3.5%)
	- chronic glomerulonephritis	n = 4 (4.5%)
	- chronic pyelonephritis	n = 1 (1.1%)
	- diabetic nephropathy	n = 4 (4.5%)
	-medullary cystic kidney disease	n = 1 (1.1%)
	-chronic kidney disease of unknown etiology	n = 2 (2.2%)

Numerical variables with normal distribution are presented as mean value ± standard deviation, while variables with non-normal distribution are presented as median values and range interval.

Five valid ARFI elastography measurements were obtained in all subjects by both operators and the median value of the five valid measurements obtained in one kidney ranged from 0.58 to 4.14 m/s. The number of failed measurements ranged between 0 and 5 for one kidney.

The kidney shear wave speed values (m/s) obtained in the right vs. left kidney were similar for both operators: 2.45±0.84 (right kidney-operator 1) vs. 2.42±0.87 (left kidney- operator 1), p = 0.80 and 2.35±0.73 (right kidney- operator 2) vs. 2.29±0.73 (left kidney- operator 2), p = 0.54.

A strong agreement was obtained between kidney shear wave speed measurements in the right and left kidney obtained by the two operators: ICC = 0.71 (95% CI: 0.53–0.83) and ICC = 0.69 (95% CI: 0.48–0.81), respectively.

Because the kidney shear wave speed values assessed by ARFI elastography were similar in the two kidneys, for both operators, and because ICC were similar for the right and left kidney, we decided to study the reproducibility of ARFI elastography measurements only in one kidney and we chose the right kidney.

Smaller ICCs were obtained in “healthy subjects” as compared to patients with kidney diseases, in women as compared with men, in subjects younger than 50 years as compared with those aged at least 50 years, in obese subjects as compared with normal weight and overweight subjects, and in case of measurements depth <4 cm or >6 cm as compared with those performed at a depth of 4–6 cm from the skin (Table 2).

**Table 2:** Influence of different factors on the inter-operator reproducibility of kidney shear wave speed measurements assessed by means of ARFI elastography.

Factor		Nr. ofpatients	ICC
Gender:	- men	40	0.78 (95% CI: 0.53–0.83)
	- women	48	0.59 (95% CI: 0.22–0.79)
Age:	- <50 years	45	0.63 (95% CI: 0.29–0.82)
	- ≥50 years	43	0.71 (95% CI: 0.41–0.87)
BMI:	- <25 kg/m^2^	44	0.66 (95% CI: 0.33–0.84)
	- 25–29.9 kg/m^2^(overweight subjects)	29	0.78 (95% CI: 0.51–0.90)
	- ≥30 kg/m^2^(obese subjects)	15	0.36 (95% CI: 0.19–0.76)
Kidneypathology:	- without kidneypathology	68	0.68 (95% CI: 0.45–0.82)
	- with kidneypathology	20	0.75 (95% CI: 0.30–0.93)
Measurementdepth	<4 cm	22	0.32 (95% CI: 0.129–0.595)
	4–6 cm	29	0.81 (95% CI: 0.605–0.919)
	>6 cm	16	0.60 (95% CI: 0.177–0.863)

The mean kidney shear wave speed values obtained by the two operators were similar regarding the age, gender, BMI, measurement depth or the presence or absence of renal pathology (Table 3).

**Table 3:** Influence of different factors on the mean kidney shear wave speed values obtained by means of ARFI elastography by the two operators (a *p* value *<*0.05 was considered as statistically significant and are in bold marked).

Factor		Nr of patients		Kidney shear wavespeed values (m/s)		p
				Operator 1	Operator 2	Operator 1 vs 2
Gender:	men	40		2.15±0.66	2.19±0.86	0.82
	women	48		2.51±0.76	2.66±0.77	0.33
	p (men vs women)			**0.02**	**0.009**	
Age:	- <50 years	45		2.53±0.65	2.71±0.74	0.22
	- ≥50 years	43		2.13±0.78	2.17±0.89	0.81
	p (<50 vs ≥50 years)			**0.008**	**0.002**	
BMI:	<25 kg/m^2^		44	2.37±0.68	2.57±0.87	0.22
	25–29.9 kg/m^2^ (overweight subjects)		29	2.14±0.80	2.18±0.87	0.85
	≥30 kg/m^2^ (obese subjects)		15	2.68±0.63	2.59±0.61	0.70
	p (normal vs overweight):			0.08	**0.04**	
	p (normalweigth vs obese):			0.12	0.93	
	p (overweight vs obese):			**0.02**	0.11	
Kidneypathology:	without kidney pathology		68	2.42±0.70	2.54±0.83	0.35
	with kidney pathology		20	2.11±0.79	2.14±0.84	0.92
	p (without vs with pathology):			0.11	0.06	
Measurementdepth	<4 cm		22	2.61±0.78	2.29±0.64	0.15
	4–6 cm		29	2.20±0.87	2.23±0.90	0.90
	>6 cm		16	2.18±0.85	2.17±0.59	0.95
	p (<4 vs 4–6 cm)			**0.04**	0.79	
	p (<4 vs>6 cm)			0.06	0.55	
	p (4–6 vs>6 cm)			0.94	0.81	

The mean kidney shear wave speed values (m/s) were higher, but not statistically significant, in subjects without known kidney pathology as compared with those with kidney diseases for both operators: 2.42±0.70 vs. 2.11±0.79, p = 0.11 (operator 1) and 2.54±0.83 vs. 2.14±0.84, p = 0.06 (operator 2).

## Discussion

Regarding the use of ARFI elastography in renal tissue assessment, published data is scarce, despite the possible usefulness of the method in establishing a correlation with renal fibrosis [18]. It seems that kidney elastography shows higher values than normal liver and pancreas, but with a high standard deviation [34], [35]. Most studies using ARFI elastography on kidneys were performed on transplanted kidneys [17], [18], [36].

As already mentioned in the introduction, before proceeding to validate this method and its extensive use in different pathologies in the non-transplanted kidney, it is important to demonstrate that this method is reproducible, as it has already been proven regarding assessing liver fibrosis [37], [38].

Our study is the first report regarding the factors which influence the reproducibility of kidney shear wave speed measurements by means of ARFI elastography in non-transplanted kidney.

We obtained a strong inter-operator agreement between ARFI elastography measurements performed in the kidney (ICC = 0.71 in the right kidney and 0.69 in the left kidney). The inter-operator agreement obtained between kidney shear wave speed measurements is lower than the one reported in published studies regarding liver stiffness (LS) measurements by means of ARFI elastography, in which the ICCs ranged between 0.81 [38] and 0.86 [37]. This difference could be explained by the fact that the kidney is usually more difficult to approach due to its position and size, as compared to the liver. Another possible explanation could be the fact that, while both operators who performed ARFI elastography measurements were experienced in the field of abdominal ultrasonography, only one (the gastroenterologist) had a large experience in the field of ARFI elastography.

However, the inter-operator agreement was stronger than those reported by Syversveen et al. [36] and Ozkan et al. [19] on transplanted kidneys, where the ICCs ranged between 0.31 [36] and 0.47 [19], and from that reported by Guo et al [39] which reported an ICC of 0.6 in a cohort of 40 healthy volunteers. Also in these studies two operators were used in order to assess the ARFI elastography reproducibility for kidney shear wave speed. An explanation of the significantly higher ICCs reported in our study as compared with the studies of Syversveen et al. [36] and Ozkan et al. [19] is the fact that we assessed non-transplanted patients and there may be a difference between the reproducibility of kidney shear wave speed in transplanted and non-transplanted kidney. Regarding our better ICCs in comparison with the study published by Guo et al [39] which assessed also non-transplanted patients an explanation can be the experience in ultrasound or the different races of the patients (European in our study and Asian in the other study).

The strong inter-operator agreement obtained in our study could suggest that an experienced operator in standard ultrasound examination can perform kidney shear wave speed measurements by means of ARFI elastography, after a short training.

We found that the following subgroups were associated with smaller ICCs: female gender, subjects without renal diseases, age under 50, obesity and measurement depth 4–6 cm from the skin.

Regarding the influence of BMI on kidney shear wave speed reproducibility by means of ARFI elastography, we obtained good and very-good inter-operator agreements in normal weight and overweight subjects (ICC = 0.66 and 0.78, respectively), while in obese subjects the inter-operator agreement was fair (ICC = 0.36), but it must be noted that the number of obese subjects included in our study was smaller than that of normal weight and overweight ones. The explanation for the lower ARFI elastography reproducibility in obese patients is probably linked to the quality of acoustic window which, as known, is not very good in obese subjects. These data are similar with those reported in studies which evaluated the reproducibility of liver stiffness (LS) measurements by means of ARFI elastography [38] or TE [40].

We obtained a better reproducibility of kidney shear wave speed measurements in men as compared with women, these results being similar with those reported in studies which evaluated the reproducibility of LS measurements by means of ARFI elastography [38]. Gender related differences in BMI cannot explain this difference of ARFI elastography reproducibility between men and women, because the percentage of obese subjects was higher in men than in women: 25% vs. 10.4%. An explanation could still be the abdominal fat, which usually is larger in women than in men, but in our study we did not measure the abdominal waist circumference.

Regarding the influence of kidney pathology on ARFI elastography reproducibility, we observed a good inter-operator agreement in both “normal subjects” and kidney pathology subjects, with slightly better results in patients with kidney diseases: ICC = 0.75 vs. ICC = 0.68. Even if the number of patients with kidney pathology was lower than that of “healthy subjects”, this is an important finding because in order to use this elastographic method in clinical practice, it is very important to be reproducible in patients with kidney diseases.

In our present study we observed a better reproducibility of kidney shear wave speed assessed by ARFI elastography in subjects older than 50 years, as compared with those younger than 50 (ICC = 0.71 vs. 0.63). This could be due to the fact that the percentage of females was higher in subjects younger than 50, as compared with those aged at least 50 years: 68.8% vs. 41.4% (p = 0.01). It is possible that this higher percentage of women, in whom the inter-operator agreement was lower, outweighs the higher (even if not significantly) percentage of obese subjects from the subgroup of subjects over 50: 21.9% vs. 11.9% (p = 0.33), thus leading to a stronger inter-operator agreement in older subjects.

We observed that the best reproducibility of kidney shear wave speed was obtained for ARFI elastography measurements performed at 4–6 cm from the skin. It should be specified that also for LS evaluation by ARFI elastography, published studies [41] demonstrated a lower performance of this technique when the measurements are performed superficial or very deep (there wasn’t any depth adjustment on the imaging machine).

In our present study, the trend of kidney shear wave speed values assessed by ARFI elastography was to decrease in patients with known renal pathology, but without reaching statistical significance, probably because of the relatively small number of patients with renal pathology included in the study. This is an interesting finding, different from the results of liver elastography and in line with the recently published data by Guo et al [39] and also by our group [42] which evaluated the kidney shear wave speed by ARFI elastography in healthy volunteers and patients with different impairment of renal function. These data suggest that factors other than interstitial fibrosis have an important role in kidney shear wave speed measurements, one of them being renal blood flow [26].

The kidney shear wave speed values obtained by the two operators in “normal” subjects were higher than that presented by Gallotti et al [34] and Guo et al [39]: 2.42 m/s and respectively 2.54 m/s vs. 2.24 m/s and 2.15 m/s. One explanation for these different results can be the different demographic parameters or different depth of ARFI elastography measurements. Also, compared with Guo et al study [39], the race of our subjects was different. It should be specified that in chronic hepatitis C patients the LS values assessed by ARFI elastography for the same stage of histological fibrosis were significantly different between Caucasian and Asian patients [43].

Our study has some limitations including: relatively small number of patients with kidney disease and also the lack of renal biopsy in these patients, the inclusion of healthy volunteers as “normal subjects” without renal tests or the lack of analyzing the role of hydration or urinary bladder filling on the reproducibility of kidney shear wave speed measurements or on the kidney shear wave speed values assessed by ARFI elastography in subjects with or without renal diseases. But, regarding the information about hydration or urinary bladder filling, these data were lacking in other published studies, which analyzed the kidney shear wave speed in various categories of subjects, as well [34], [36], [39]. Also, in our study we did not analyze the influence of the region of interest position, all the measurements being performed in the mid-portion of the kidney, in the renal cortex, but not at the renal poles. In a previous study performed by our group we showed that the rate of success when trying to perform the measurements in the renal poles was low compared to the mid-portion of the renal parenchyma [27], possibly because of renal anisotropy, and therefore we did not attempt to measure the reproducibility of the method in this region. Regarding this limitation, it should be specified that this did not influence the comparison of our results with those published by Gallotti et al [34] and Guo et al [39], because the same region of interest position was used. Another limitation is the analysis of the influence of measurement depth only in 76.1% of the subjects, because it was not available in all subjects in the database, but even in this cohort interesting and useful results were found (best ARFI elastography reproducibility in the measurements performed at 4–6 cm from the skin). In our study we used only two operators in order to assess the interoperator reproducibility of kidney shear wave speed and this can be also an other limitation of our results. But, it should be specified that also other studies which analyzed the interoperator reproducibility of kidney shear wave speed [19], [36], [39] and LS [37], [38], [44], [45] used also only two operators.

In **conclusion**, our study demonstrated that ARFI elastography is a reproducible non-invasive method for kidney shear wave speed evaluation. However, ARFI elastography reproducibility was lower in women, “healthy subjects”, subjects younger than 50 years and especially in obese subjects and measurements performed 4 cm from the skin or 6 cm below the skin. The trend of kidney shear wave speed values assessed by ARFI elastography was to decrease with renal function impairment, but future studies are needed in this field.
